# Experimental and Clinical Pharmacology of *Andrographis paniculata* and Its Major Bioactive Phytoconstituent Andrographolide

**DOI:** 10.1155/2013/846740

**Published:** 2013-03-24

**Authors:** Thanasekaran Jayakumar, Cheng-Ying Hsieh, Jie-Jen Lee, Joen-Rong Sheu

**Affiliations:** ^1^Department of Pharmacology, Taipei Medical University, Taipei 110, Taiwan; ^2^Graduate Institute of Medical Sciences, Taipei Medical University, 250 Wu-Hsing Street, Taipei 110, Taiwan; ^3^Department of Surgery, Mackay Memorial Hospital, Taipei 104, Taiwan; ^4^Department of Nursing, Mackay Medicine, Nursing and Management College, Taipei 112, Taiwan

## Abstract

*Andrographis paniculata* (Burm. F) Nees, generally known as “king of bitters,” is an herbaceous plant in the family Acanthaceae. In China, India, Thailand, and Malaysia, this plant has been widely used for treating sore throat, flu, and upper respiratory tract infections. Andrographolide, a major bioactive chemical constituent of the plant, has shown anticancer potential in various investigations. Andrographolide and its derivatives have anti-inflammatory effects in experimental models asthma, stroke, and arthritis. In recent years, pharmaceutical chemists have synthesized numerous andrographolide derivatives, which exhibit essential pharmacological activities such as those that are anti-inflammatory, antibacterial, antitumor, antidiabetic, anti-HIV, antifeedant, and antiviral. However, what is noteworthy about this paper is summarizing the effects of andrographolide against cardiovascular disease, platelet activation, infertility, and NF-**κ**B activation. Therefore, this paper is intended to provide evidence reported in relevant literature on qualitative research to assist scientists in isolating and characterizing bioactive compounds.

## 1. Introduction

*Andrographis paniculata* (Burm. F) Nees, commonly known as the “king of bitters,” is an herbaceous plant belonging to the Acanthaceae and is found throughout tropical and subtropical Asia, Southeast Asia, and India. In India, *A. paniculata* is known as “Kalmegh”; in China it is known as “Chuan-Xin-Lian”; in Thailand it is known as “Fah Tha Lai”; in Malaysia it is known as “Hempedu bumi”; in Japan it is known as “Senshinren”; and in Scandinavian countries it is known as “green chiretta” [1]. Extracts of this plant and andrographolide exhibit pharmacological activities such as those that are immunostimulatory [1, 2], antiviral [3], and antibacterial [4]. As major active constituent, andrographolide exhibits a broad range of biological activities, such as anti-inflammatory, antibacterial, antitumor, antidiabetic, antimalarial, and hepatoprotective [5]. Because of the impressive variety of these biological activities, researchers propose obtaining various leads by structurally modifying andrographolide. In recent decades, numerous andrographolide derivatives have emerged and their pharmacological activities have also been evaluated. However, studies that have comprehensively summarized or analyzed *A. paniculata* and its derivatives have been minimal. Therefore, to contribute to the advanced trends of research on andrographolide, this paper provides thorough information regarding the pharmacological activities of *A. paniculata* and its major compound andrographolide.

### 1.1. Chemical Structure

**Figure 1:**
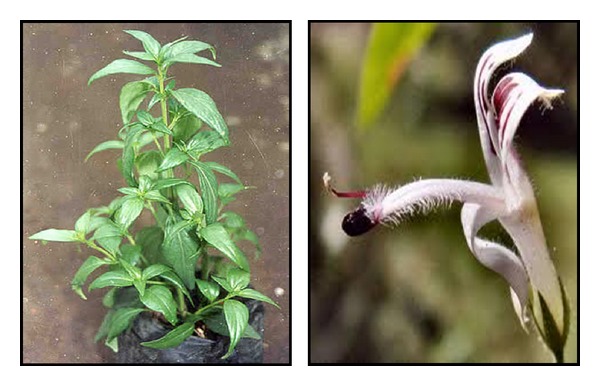
Morphology of *Andrographis paniculata*.

**Figure 2:**
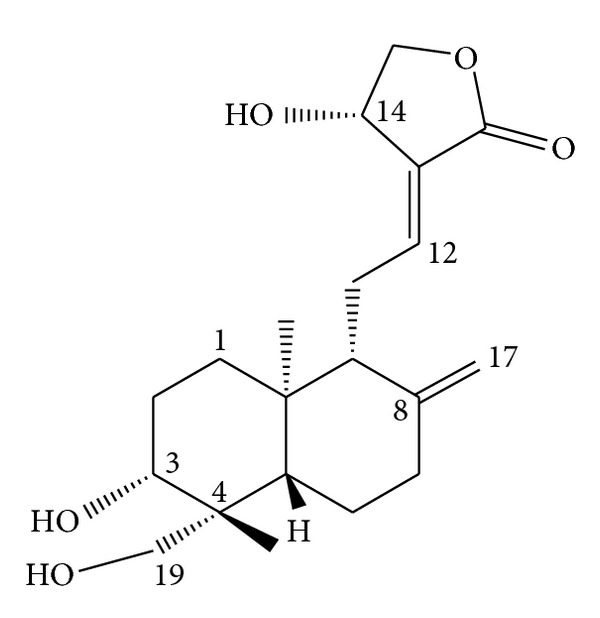
Chemical structure of andrographolide.

Andrographolide is a major bioactive phytoconstituent found in various parts of *A. paniculata *(Figure 1), but particularly in the leaves. The chemical name of andrographolide is 3*α*, 14, 15, 18-tetrahydroxy-5*β*, 9*β*H, 10*α*-labda-8, 12-dien-16-oic acid *γ*-lactone (Figure 2), and its molecular formula and weight are C_20_H_30_O_5_ and 350.4 (C 68.54%, H 8.63%, and O 22.83%), respectively. The structure of andrographolide has been analyzed by using X-ray, 1H,13 C-NMR, and ESI-MS [6–10]. Although andrographolide is not very soluble in water, it is soluble in acetone, chloroform, ether, and hot ethanol. Crystalline andrographolide was reported to be highly stable, over a period of three months [11]. Rajani et al. [8] reported a simple and rapid method for isolating andrographolide from the leaf of *A. paniculata.* They extracted it using a 1 : 1 mixture of dichloromethane and methanol and then isolated the andrographolide directly from the extract by performing recrystallization. The purity of the compound has been evaluated with thin-layer chromatography (TLC), UV absorption spectrum, high-performance liquid chromatography (HPLC), liquid chromatography-mass spectrometry (LCMS), and differential scanning calorimetry (DSC), which revealed the melting point of andrographolide to be 235.3°C [8, 9].

### 1.2. Biological Activities of Andrographolide

Andrographolide has been reported to have a wide range of biological activities, such as those that are anti-inflammatory [12], antiallergic [13], antiplatelet aggregation [14, 15], hepatoprotective [16], and anti-HIV [17]. In addition to these activities, the ability of ethanol or an aqueous extract of *A. paniculata* to decrease blood glucose levels in normal rats or streptozotocin diabetic rats has been documented [18]. In biological systems, andrographolide can interact with many inter- and intracellular constituents as a bipolar compound, thus ensuing in many biological responses. A recent study demonstrated that *A. paniculata* polysaccharides combined with andrographolide can ease the recovery of diabetic nephropathy [18].

## 2. Experimental Studies

### 2.1. Effects on Antioxidant Defense

Antioxidant defense systems may only partially prevent oxidative damage [19]. Hence, there is interest in using dietary supplements containing antioxidants to protect the components of the human body from oxidative damage. Currently, the most commonly used synthetic antioxidants are butylated hydroxyanisole (BHA), butylated hydroxytoluene (BHT), propyl gallate, and *tert*-butylhydroquinone. However, BHA and BHT have restricted use in foods because they are suspected to be carcinogenic and to cause liver damage [20]. Therefore, there is growing interest in using natural additives as potential antioxidants [21, 22].

Several studies have reported the antioxidant activities of *A. paniculata *and its constituents. Verma and Vinayak [23] reported that the aqueous extract of* A. paniculata *significantly increased the activities of antioxidant defense enzymes such as catalase, superoxide dismutase, and glutathione-S-transferase and reduced glutathione content. The extract significantly inhibits lipid peroxidation by lowering the levels of thiobarbituric-acid-reactive substances in the liver and kidney of diabetic rats (as compared to normal rats) and also significantly increases the level of hepatic glutathione concentrations [24]. A pretreatment of andrographolide was reported to significantly attenuate the accumulation of the phorbol-12-myristate-13-acetate- (PMA-) induced formation of ROS and N-formyl-methionyl-leucyl-phenylalanine-(fMLP-) inducing adhesion of rat neutrophils [25]. Andrographolide exhibited free radical-scavenging ability, thus reduced oxidative stress and thiobarbituric-acid-reactive substance formation [26].

### 2.2. Anti-Inflammatory Effects

Andrographolide has been reported to significantly reduce the inflammation caused by histamine, dimethyl benzene, and adrenaline [27]. Overproduction of NO and prostaglandin E2 (PGE2), because of the expression of inducible isoforms of nitric oxide synthase (iNOS) and cyclooxygenase-2 (COX-2), plays a significant role in the inflammatory processes of activated macrophages. The secretion of proinflammatory cytokines from macrophages stimulated and promoted by lipopolysaccharide, which causes induction of iNOS, results in increased production of NO. The methanol extract of *A. paniculata* and andrographolide incubated with macrophages have been reported to inhibit LPS-stimulated NO production in a concentration-dependent manner [28, 29]. Chiou et al. [30] observed that andrographolide inhibits lipopolysaccharide-induced nitric oxide (NO) production and inducible NO synthase (iNOS) expression in the murine macrophage-like cell line RAW 264.7. Administering andrographolide to rats fully restored the maximal contractile response of the thoracic aorta to phenylephrine after incubation with LPS and alleviated the decrease in the mean arterial blood pressure of anesthetized rats. Andrographolide has also been reported to suppress IL-2 production and T-cell proliferation in a mixed lymphocyte reaction and to inhibit dendritic cell maturation and antigen presentation [31].

### 2.3. Anticancer Activity

Natural products are recognized as sources for drugs used to treat several human ailments including cancers. Vincristine, irinotecan, etoposide, and paclitaxel are examples of many natural pharmaceuticals derived from plants [32]. Despite the discovery of numerous drugs of natural origin, searching for new anticancer agents is still necessary to provide drugs that are less toxic and more effective and to increase their variety and availability. Samples with pharmacological usage should be accounted for when selecting plants to treat cancer because several ailments reflect disease states bearing relevance to cancer or cancer-like symptoms [33]. Andrographolide exhibited potent cytotoxic activity against KB (human epidermoid leukemia) and P388 (lymphocytic leukemia) cells [34]. Among the diterpenoid lactones isolated from the ethyl acetate fraction of *A. paniculata*, andrographolide had strong anticancer activity by inducing cell differentiation in mouse myeloid leukemia cells [35]. Andrographolide was found to inhibit the proliferation of various cell lines including leukemia, breast cancer, lung cancer, and melanoma cells [2, 36]. Furthermore, this compound has strong anticancer activity against human colorectal carcinoma LoVo cells by inhibiting cell cycle progression [37]. A potent growth inhibitory effect of andrographolide has been demonstrated in acute promyelocytic leukemic cells (HL-60 and NB4) that are mediated by inducing cell differentiation and apoptosis [38, 39]. Andrographolide was also reported to suppress the adhesion of gastric cancer cells which express high-level sialyl Lewis X to human vascular endothelial cells by blocking E-selectin expression and, thus, may represent a candidate therapeutic agent for cancer [40]. Lim et al. [41] demonstrated that the anticancer mechanisms for andrographolide include the inhibition of Janus tyrosine kinases-signal transducers and activators of transcription, phosphatidylinositol 3-kinase and NF-*κ*B signalling pathways, suppression of heat shock protein 90, cyclins and cyclin-dependent kinases, metalloproteinases and growth factors, and the induction of tumour suppressor proteins p53 and p21, leading to the inhibition of cancer cell proliferation, survival, metastasis, and angiogenesis.

*In vivo* models of the anticancer activity of andrographolide have been used against MCF-7 and HT-29 tumor xenografts and B16F0 melanoma [38]. In a radiation therapy study, andrographolide was found to sensitize Ras-transformed cells and significantly delay tumor growth [42]. Sheeja and Kuttan [43] demonstrated that *A. paniculata* extract or andrographolide alone could stimulate cytotoxic T lymphocyte production through the enhanced secretion of IL-2 and IFN-c by T cells, thereby inhibiting tumor growth *in vivo*. Inhibition of angiogenesis is currently perceived as a promising strategy in treating cancer. In a significant invention, *A. paniculata* and andrographolide alone were found to inhibit tumor-specific angiogenesis by regulating the production of various pro- and antiangiogenic factors, such as pro-inflammatory cytokines, NO, vascular endothelial growth factor, IL-2, and the tissue inhibitor of metalloproteinase-1 [43]. A recent study demonstrated that andrographolide inhibits breast cancer cell proliferation, migration, and cell cycle arrest at the G2/M phase and induces apoptosis through a caspase-independent pathway. Their experimental evidence suggests that andrographolide attenuates endothelial cell motility and tumor-endothelial cell interaction [44]. The antitumor activity of andrographolide in an *in vivo* model was correlated with the downregulation of PI3 kinase/Akt activation, inhibition of proangiogenic molecules, such as OPN, and VEGF expressions [44].

### 2.4. Immunomodulatory Activity

Purified andrographolide (1 mg/kg body weight) or intragastric administration of ethanol extracts of the stems and leaves (25 mg/kg body weight) to mice stimulate antibody production and the delayed-type hypersensitivity response to sheep red blood cells [45]. The extract and purified andrographolide were also reported to stimulate an innate immune response in mice, which was measured according to the macrophage migration index, phagocytosis of leucine-labelled *Escherichia coli*, and proliferation of splenic lymphocytes stimulated by *A. paniculata *extract [45]. The immunomodulatory property of a diterpene lactone andrographolide was reported to be associated with the enhancement of the proliferation of human peripheral blood lymphocytes, as well as the production of key cytokines and the expression of Y Xu 21 immune activation markers in whole blood cells in culture *in vitro* [46]. Rajagopal et al. [2] and Kumar et al. [1] have reported the immunostimulatory activity of andrographolide *in vitro* in PHA-stimulated human peripheral blood lymphocytes (HPBLs) by increased proliferation of lymphocytes and production of IL-2. *In vivo *immune responses, such as an antibody response to a thymus-dependent antigen and delayed-type hypersensitivity, were considerably lessened in mice treated with andrographolide. In addition, Iruretagoyena et al. [47] reported that andrographolide enhanced the tolerogenic properties of immature dendritic cells (DCs) in experimental autoimmune encephalomyelitis (EAE) by inhibiting NF-kappa B activation in murine DCs. Andrographolide was also reported to reduce IFN-*γ* and IL-2 production in murine T cells stimulated with concanavalin A (Con A) *in vitro* [48]. Moreover, andrographolide was reported to inhibit the production of TNF-*α* and IL-12 in macrophages stimulated by lipopolysaccharide [49].

### 2.5. Hepatoprotective Activity

Liver diseases of various origins remain a serious health problem and a major cause of mortality. In the absence of reliable hepatoprotective drugs in modern medicine, herbs and plants play a vital role in managing several liver disorders [50, 51]. Extensive literature related to the hepatoprotective activity of molecules from herbal sources shows that there is a vast array of molecules exhibiting potent hepatoprotective efficacy. The Indian systems of medicine have long used *A*. *paniculata *as a hepatostimulant and hepatoprotective agent [16]. *A*. *paniculata* is also an ingredient in several polyherbal preparations used as hepatoprotectants [52], one of which has been reported to be efficacious in chronic hepatitis B viral infection [53]. A recent study showed that andrographolide attenuated concanavalin A-induced liver injury and inhibited hepatocyte apoptosis [54]. Shukla et al. [55] reported observing choleretic effects of andrographolide in conscious rats and anesthetized guinea pigs. The effect of andrographolide was found to be more potent than silymarin against acetaminophen-induced reduction of the volume and contents of bile. Andrographolide was also shown to protect against ethanol-induced hepatotoxicity in mice with an equivalent efficacy of silymarin [56]. Oral pre- and posttreatments of adult rats with an extract of *A. paniculata *were protective against an ethanol-induced increase in serum transaminases. A protective effect of a single oral dose each of the extract and of andrographolide has been studied in carbon tetrachloride- (CCl_4_-) induced hepatic microsomal lipid peroxidation. Rana and Avadhoot [57] reported the hepatoprotective effects of the crude alcohol extract of leaves against CCl_4_-induced liver damage; these effects have had also been established against paracetamol-induced toxicity in an *ex vivo *rat model of isolated hepatocytes [58]. Plant extracts of *A. paniculata* showed hepatoprotective characters consistent with the folk use and pharmacology [59]. 

### 2.6. Antimicrobial Effects

Antimicrobial drugs have caused a dramatic change not only in the treatment of infectious diseases but to the fate of mankind. Antimicrobial chemotherapy has made noteworthy advances, resulting in positive observations that infectious diseases might be dominated in the near future. However, in reality, emerging and reemerging infectious diseases have indicated a countercharge from infections. Infections with drug-resistant organisms hang back an imperative problem in clinical practice that is complicated to explain. If an unsuitable antimicrobial agent is preferred over the treatment of infection with drug-resistant microorganisms, the therapy may not achieve beneficial effects and may lead to a worse prognosis. *A. paniculata* and andrographolide have been reported to exhibit potent antimicrobial activity against various microbial organisms.

*In vitro *antibacterial activity of the crude powder of* A. paniculata* has been reported against *Salmonell*a, *Shigella, E. coli*, gram A streptococci, and *Staphylococcus aureus*, even at a concentration of 25 mg/mL. Singha et al. [4] found significant antibacterial activity in an aqueous extract with andrographolide. A similar result was found in a crude aqueous extract of leaves that exhibit significant antimicrobial activity against gram-positive *S. aureus*, methicillin-resistant *S. aureus*, and gram-negative *Pseudomonas aeruginosa* [60]. Significant activity against enterohemorrhagic strains of *E. coli* was found in the ethanol extract of *A. paniculata* [61]. The virucidal activity of andrographolide has been reported against herpes simplex virus 1 (HSV-1) without having any significant cytotoxicity [62]. At a concentration of 0.05 mg/mL of a chloroform extract of *A. paniculata,* the plant completely inhibits malarial parasitic growth within 24 h of incubation; and the same inhibition has been noted within 48 h with methanol extract concentration of 2.5 mg/mL [63]. A methanol extract was found to inhibit *Plasmodium falciparum *substantially at a 50% inhibitory concentration (IC50) of 7.2 *μ*g/mL [64]. The ethanolic extract of *A. paniculata* was effective against upper respiratory tract infection [65]. The antimicrobial activity of *A. paniculata *against nine bacterial strains, *Salmonella typhimurium, E. coli, Shigella sonnei, Staphylococcus aureus, Pseudomonas aeruginosa, Streptococcus pneumonia, Streptococcus pyogenes, Legionella pneumophila, *and *Bordetella pertussis,* has also been reported [66].

### 2.7. Antiviral Effects

The antiviral activities of plant extracts have been renewed and have been the topic of passionate scientific investigation. Several medicinal plant extracts have shown antiviral activities against some RNA and DNA viruses. Among these plants is *A. paniculata *which exhibits a neutralizing activity against the human immunodeficiency virus (HIV) [67]. Andrographolide was investigated for antiviral activity against herpes simplex virus (HSV) [62, 68], HIV [3], flaviviruses, and pestiviruses [69]. Lin et al. [70] demonstrated that 25 *μ*g/mL of ethanolic extract of *A*. *paniculata *and 5 *μ*g/mL of andrographolide effectively inhibit the expression of Epstein-Barr virus (EBV) lytic proteins, Rta, Zta, and EA-D, during the viral lytic cycle in P3HR1 cells. A recent study has demonstrated that *A. paniculata* has the most antiviral inhibitory effects among six medicinal plants tested against DENV1-infected Vero E6 cells [71]. 

### 2.8. Antipyretic and Analgesic Effects

In Asian countries, *A. paniculata *has been widely used for its antipyretic, analgesic, protozoacidal, antihepatotoxic, anti-HIV, immunostimulant, anticancer effects [36]. It had been reported that andrographolide, with oral doses of 100 and 300 mg/kg, produced a significant antipyretic effect after 3 h administration of brewer's yeast-induced fever in rats [72]. In addition, doses of 180 or 360 mg/kg of andrographolide were also found to relieve fever in humans by the third day after administration [73]. Madav et al. [72] have also reported that 300 mg/kg of andrographolide, administered orally, had significant analgesic activity on acetic-induced writhing in mice and on the Randall-Selitto test in rats, but without any effect on the hot plate test in mice. These authors have also reported that intraperitoneal administration of 4 mg/kg of andrographolide exhibited an analgesic effect, whereas the former study, 300 mg/kg administered orally did not. The different routes of administration between these experiments could contribute to this discrepancy [72].

### 2.9. Antimalarial Effects

*In vitro* and *in vivo* studies performed by Rahman et al. [63] showed that *A. paniculata *produced significant antimalarial effects. Chloroform extract of this plant shows better effect than the methanol extract because it showed complete parasite growth inhibition as low as 0.05 mg/mL drug dose within 24 h incubation period as compared to methanol extract of drug dose of 2.5 mg/mL but under incubation time of 48 h of the same plant species. *In vivo* activity of *A. paniculata *also demonstrated higher antimalarial effect [63]. Fractions isolated from *A. paniculata *also exhibited antimalarial activity [74]. Misra et al. [75] have isolated andrographolide, neoandrographolide, deoxyandrographolide and andrographolide from the leaves of *A. paniculata* that showed anti-malarial activity against *Plasmodium berghei *NK65 in *Mastomys natalensis*.

### 2.10. Larvicidal and Ovicidal Effects

Plant products have been used by traditionally human communities in many parts of the earth against the vectors and species of insects. The phytochemicals derived from plant sources can act as larvicides, insect growth regulators, repellents, and ovipositional attractants and have deterrent actions as observed by many researchers [76–80]. The treatment of different products of *A. paniculata* greatly affected the larval growth of *Anopheles stephensi* and caused malformation and mortality in a dose-dependent manner [81]. An ethanolic extract of *A. paniculata* caused moderate ovicidal activity against various age groups of *Aedes stephensi*, but it inflicted delayed effects such as high larval, pupal, and adult mortality, thereby suppressing the vector population and adversely influencing transmission of the disease pathogen [82]. The leaf extract of *A. paniculata* with different solvents of benzene, hexane, ethyl acetate, methanol, and chloroform exhibited larvicidal and ovicidal activities against *Culex quinquefasciatus* Say and *Aedes aegypti* L., whereas ethyl acetate and methanol extracts of the plant showed only ovicidal activity against *Culex quinquefasciatus* and *Aedes aegypti* [83]. They have also found 100% mortality against two mosquito species exerted by ethyl acetate and methanol extracts of the plant. A recent study performed by Sheeja et al., 2012 suggest that the leaf extracts of *A. paniculata* may have the potential to be used as an ideal eco-friendly approach for the control of the filarial vector *Culex quinquefasciatus *[76].

### 2.11. Renoprotective Effects

The recurrence of urolithiasis is critical; thus, preventing and treating stone formation are highly recommended. The most recent data suggest that 27 million people have chronic kidney disease, representing nearly one in seven adults and a 30% increase over the past decade [84]. In the Unites States, more than 200 thousand people suffer from kidney failure. A similar increase in the incidence of end-stage renal failure caused by an increasing incidence of the risk factors for renal disease has occurred in many Asian countries [85]. A study found that the aqueous extract of *A. paniculata* could considerably alleviate the nephrotoxic action of gentamicin in male albino rats, thus exhibiting marked renoprotective activity [86].

### 2.12. Antifertility Effects

Efforts are underway to develop antifertility products from plants. Many plants are reported to have fertility-regulating properties in ancient Indian literature [87]. Numerous plants have been tested for their antifertility activities in laboratory animals [88, 89] and several animal studies have reported an effect of *A. paniculata *on male and female reproduction. Early reports of oral administration of the powdered stem of *A. paniculata* have shown an antifertility effect on male Wistar mice, but no impact on fertility in female mice [90]. It has also been reported that administering *A. paniculata *results in abortion in pregnant rabbits. Moreover, the herb is reported to suppress the growth of human placental chorionic trophoblastic cells in vitro [91]. Zoha et al. [92] reported feeding sun-dried *Andrographis* powder to female mice at a dose of 2 g/kg bw/day for 6 weeks and then mated them with untreated males of proven fertility, thus inhibiting pregnancy in 100% of the tested animals. Oral administration of *Andrographis paniculata *extract during the first 19 days of pregnancy in doses of 200, 600, and 2000 mg/kg did not exhibit any effect on the elevated level of progesterone in the blood plasma of rats [46]. Animal studies have also shown that *A. paniculata* may have contraceptive or antifertility effects following long-term treatment at high doses (20 mg/rat) [93]. However, there was a large degree of discrepancy in the results, with some studies demonstrating no untoward effects even at the 1000 mg/kg dose [48]. Administering dry leaf powder to male albino rats (20 mg daily for 60 days) has been shown to inhibit spermatogenesis, degenerative changes in the seminiferous tubules, regression of Leydig cells, and regressive or degenerative changes in the epididymis, seminal vesicle, ventral prostate, and coagulating glands [94]. Andrographolide also produced similar results when orally administered to male Wistar albino rats for 48 days. A study reported no toxicity of andrographolide (50 mg/kg) treatment for up to 8 weeks in the number and motility of sperm [95]. It was reported that the effect of andrographolide or *A. paniculata* on sex hormones in patients with an impaired testosterone level might be able to return hormone levels to normal and treat decreased libidos and decreased mental and physical sexual activity. 

### 2.13. Antihyperglycemic Activities

Diabetic nephropathy has become the leading cause of end-stage renal disease in developed countries, thus creating an increasing clinical problem [96]. To prevent and treat diabetic nephropathy, current methods using agents such as angiotensin-converting enzyme inhibitors, angiotensin-II receptor blockers, and antihypertensive drugs have been attempted in clinical practice [97]. Despite these treatments, numerous patients still develop intractable diabetic nephropathy. This has prompted considerable interest in using traditional medicines to treat this condition. Orally administered glucose-induced hyperglycemia in nondiabetic rabbits was reported to be prevented by the extract of *A. paniculata. *Six weeks of chronic administration of the extract showed no effect on fasting blood glucose levels [98]. The ethanolic extract of *A. paniculata* at a dose of 400 mg/kg body weight twice daily for 2 weeks to diabetic rats was shown to produce a 49.8% reduction in fasting serum triglyceride levels. This was reported to be greater than the 27.7% decline that was achieved with 500 mg/kg body weight twice daily for 14 days [24]. An aqueous extract (50 mg/kg body weight) administered to streptozotocin-diabetic rats resulted in a 52.9% reduction in blood glucose levels. Dry powder of the plant material significantly decreased blood glucose levels by 61.8% at a lower dose of 6.25 mg/kg body weight [99]. Comparable results were observed by Dandu and Inamdar [100] with oral administration of an aqueous extract of *A. paniculata *leaves. A dose of 400 mg/kg was found to lower the blood glucose levels of streptozotocin-induced animals and increased the activity of superoxide dismutase and catalase. Oral administration of the decoction also significantly reduced blood glucose levels in alloxan-induced diabetic rats and reduced food and water intake when compared to vehicle-treated diabetic controls [100]. Extended mean estrous cycles were reduced from 8 to 5 days in treated diabetic rats [101]. Andrographolide appears to reduce plasma glucose concentration dose-dependently in streptozotocin-induced diabetic and normal rats, with the potential effect observed in normal rats rather than in diabetic rats [102]. This is a significant difference from the water extract, which did not show a glucose-lowering effect in a study on normoglycemic rats [100].

Andrographolide also attenuates the increase in plasma glucose in response to an intravenous glucose challenge in normal rats and enhances the uptake of radioactive glucose by isolating the soleus muscle of streptozotocin-diabetic rats in a concentration-dependent manner. Repeated intravenous administration of andrographolide in diabetic rats for three days resulted in an increase in mRNA and protein levels of glucose transporter in the soleus muscle, indicating that the glucose-lowering effect of andrographolide could be caused by more effective glucose use of the skeletal muscle [102]. However, an *in vitro* experiment concluded that the hypoglycemic effect of *A. paniculata *is caused by insulin release from pancreatic cells through ATP-sensitive potassium channels, an effect that is similar to that of other insulinotropic antidiabetic agents [103]. Subramanian et al. [104] conducted *in vitro* experiments and suggested that the inhibition of alpha-glucosidase and alpha-amylase enzyme could be the mechanism by which the ethanol extract of *A. paniculata *and andrographolide produce hypoglycemic effects. Water extract seems to be a more suitable candidate for further study because it does not affect the fasting blood glucose levels of nondiabetic animals. Therefore, identifying blood glucose-lowering constituents in both water and ethanol extracts may be of value.

### 2.14. Hypolipidemic Effects

Hyperlipidemia is a crucial factor, particularly in patients with high cholesterol levels and abnormal lipoprotein metabolisms, and has a direct relationship with cardiovascular diseases [105, 106]. Hence, the research and development of new functional foods and medicines for preventing coronary heart disease are crucial. Cholesterol and other fatty substances combine in the bloodstream and are deposited in the blood vessels to form a material called plaque [107]. The increase in lipids can cause plaque to grow over time and lead to obstructions in blood flow. If an obstruction occurs in the coronary arteries, it could result in a heart attack. Furthermore, an obstruction occurring in the arteries of the brain could lead to a stroke [108]. Hence, it is critical to actively decrease blood lipid counts to prevent and cure cardiovascular and cerebrovascular diseases. A recent study thoroughly demonstrated that andrographolide has potent hypolipidemic effects and protects the cardiovascular system without significant liver damage by lowering TC, TG, HDL-TC, and LDL-TC in mice and rats [109]. Nugroho et al. [110] reported that the purified extract of andrographolide significantly (*P* < 0.05) decreased the levels of blood glucose, triglycerides, and LDL.

### 2.15. Effects on Cardiovascular Disease

*A. paniculata* has demonstrated an increase of blood-clotting time; hence, pre- and posttreatments of the extract of *A. paniculata* after surgery significantly prevent the constriction of blood vessels, thus decreasing the risk of the subsequent closing of blood vessels after angioplasty procedures [111]. Several studies have used animal models to investigate the effects of aqueous extracts and active constituents of *A. paniculata*, both before and after experimental myocardial infarction. An extract of the plant produced antihypertensive effects because it relaxed smooth muscles in the walls of blood vessels and prevented the blood vessels from constricting and limiting blood flow to the brain, heart, and other organs [112]. A time-dependent protection of rat cardiomyocytes against hypoxia injury was reported to be caused by the pretreatment of andrographolide; this effect was reported to be associated with upregulation of cellular reduced glutathione (GSH) level and antioxidant enzyme activities [113]. Awang et al. [114] demonstrated that the dichloromethane extract of *A. paniculata* significantly reduced coronary perfusion pressure by up to 24.5 ± 3.0 mm Hg at a 3 mg dose and also reduced the heart rate by up to 49.5 ± 11.4 beats/min at this dose. The arterial constriction caused by high cholesterol in the diet and by injury to the inner lining of the blood vessel was also found to be diminished by *A. paniculata* [115]. It was reported that *A. paniculata *decreased the damage of the heart muscle, when it is administered to dogs one hour after the development of myocardial infarction [116]. These findings imply the promising use of *A. paniculata* as a favorable alternative for cardiovascular therapy.

### 2.16. Inhibitory Effects on Platelet Aggregation

**Figure 3:**
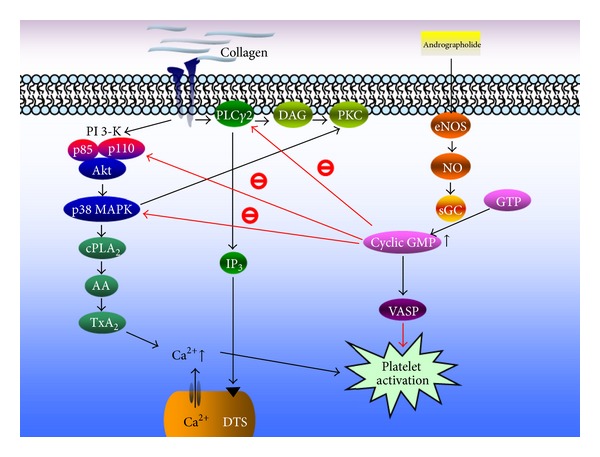
Hypothetical scheme shows the inhibitory signaling of andrographolide in platelet activation. Andrographolide can activate the endothelial nitric oxide synthase- (eNOS-) NO-cyclic GMP pathway, followed by the inhibition of both the PLC*γ*2-DAG-PKC and PI3 kinase/Akt cascades, and ultimately inhibits platelet aggregation [120].

An intravascular thrombosis is among the generators of a wide variety of cardiovascular diseases. Initiation of an intraluminal thrombosis is believed to involve platelet adherence and aggregation. Thus, platelet aggregation may play a crucial role in the atherothrombotic process [117]. Blood platelet activation and aggregation are common denominators in atherothrombotic events and various inflammatory diseases. Platelets have been viewed exclusively as mediators of thrombosis and hemostasis; their function has been extended to include prominent roles in inflammation and immunity [118]. Therefore, the use of antiplatelet agents, which can inhibit thromboembolic diseases (myocardial infarction, ischemic stroke, and vascular death) in the platelets, warrants investigation. Amroyan et al. [14] found that andrographolide inhibited PAF-induced human platelet aggregation. Moreover, Thisoda et al. [119] reported that the extract of *A. paniculata* (10–100 *μ*g/mL) significantly inhibited platelet aggregation in washed rat platelets. Our recent study demonstrated for the first time that andrographolide exhibits potent antiplatelet activity through the activation of the eNOS-NO/cyclic GMP pathway and inhibition of both the PLC*γ*2-PKC and PI3 kinase/Akt-MAPK (i.e., p38 MAPK) cascades in washed human platelets (Figure 3) [120]. Our earlier study also showed that andrographolide may involve an increase in cyclic GMP/PKG, followed by inhibition of the p38 MAPK/^•^HO-NF-*κ*B-ERK2 cascade in activated platelets. In that study, we also suggested that andrographolide may have a high therapeutic potential to treat thromboembolic disorders and may also be considered for treating various inflammatory diseases [15].

Aqueous extract, andrographolide, and DDA inhibit thrombin-induced platelet aggregation in time- and concentration-dependent manners [119]. Andrographolide inhibits platelet-activating factor- (PAF-) induced platelet aggregation in a dose-dependent manner without affecting the biosynthesis of eicosanoids. An extract of *A. paniculata *significantly inhibited *ex vivo *ADP-induced platelet aggregation in 63 patients with cardiac and cerebral vascular diseases 3 h after administration. Thirty-three of these patients, who were observed for platelet aggregation after 1 week, experienced even more significant effects. Serotonin release from platelets was significantly reduced in 20 extract-treated volunteers, although the plasma serotonin levels remained unchanged [121].

### 2.17. Inhibitory Effects on NF-kappa B (NF-*κ*B) Transcription Factors

NF-*κ*B plays a pivotal role in the pathogenesis of inflammation, prompting various drugs designed to treat human inflammatory disease to be focused on inhibiting NF-*κ*B activation [122]. Many natural compounds or herbal extracts reportedly exhibit anti-inflammatory activities that generally involve NF-*κ*B activation [123, 124]. Phytochemicals, especially flavonoids, are currently of interest because of their essential biological and pharmacological properties, including the inhibition of NF-*κ*B activation [125].

NF-kappa B comprises a family of inducible transcription factors that serve as crucial regulators of the host immune and inflammatory responses. The NF-kappa B transcription factor regulates the expression of various components of the immune system, including proinflammatory cytokines, chemokines, adhesion molecules, and inducible enzymes such as cyclooxygenase-2 and inducible nitric oxide synthase, as well as proteins that regulate the specific immune response, such as interleukin- (IL-) 2, IL-12, and interferon-*γ* that control lymphocyte proliferation and differentiation. Therefore, dysregulation of this transcription factor can lead to inflammatory and autoimmune diseases [126]. Andrographolide has been proven to attenuate inflammation by inhibiting NF-kappa B activation through the covalent modification of reduced Cys62 of p50. Mechanistically, andrographolide formed a covalent adduct with a reduced cysteine of p50, thus blocking the binding of NF-kappa B oligonucleotide to nuclear proteins. Andrographolide suppressed the activation of NF-kappa B in stimulated endothelial cells, thereby reducing the expression of the cell adhesion molecule E-selectin and prevented E-selectin-mediated leukocyte adhesion under flow [13]. Andrographolide also abrogated the cytokine- and endotoxin-induced peritoneal deposition of neutrophils, attenuated septic shock, and prevented allergic lung inflammation *in vivo*.

Other researchers have analyzed the effect of andrographolide on the activation of NF-kappa B induced by a platelet-activating factor (PAF) and N-formyl-methionyl-leucyl-phenylalanine (fMLP) in HL-60 cells differentiated to neutrophils. Andrographolide has been shown to inhibit the NF-kappa B luciferase activity induced by PAF. Andrographolide also reduced the DNA binding of NF-kappa B in whole cells and in nuclear extracts induced by PAF and fMLP. Therefore, andrographolide exerts its anti-inflammatory effects by inhibiting NF-kappa B binding to DNA, thus reducing the expression of proinflammatory proteins, such as COX-2 [127].

Several lines of evidence indicate that inhibition of NF-*κ*B transcriptional activity contributes to the protective anti-inflammatory actions of andrographolide [128, 129]. Andrographolide inhibits nuclear factor kappa B (NF-*κ*B) activation by blocking the binding of NF-*κ*B oligonucleotides to nuclear proteins [30, 128]. Recently, we demonstrated that andrographolide enhances the NF-*κ*B subunit p65 Ser536 dephosphorylation through the activation of protein phosphatase 2A in vascular smooth muscle cells [129]. We also demonstrated for the first time that andrographolide inhibited p65 Ser536 phosphorylation, reduced nuclear translocation of p65, and diminished p65 kB oligonucleotide binding in LPS/IFN-*γ*-stimulated rat VSMCs [129]. In addition, PP2A may contribute to these actions of andrographolide in rat VSMCs.

## 3. Clinical Studies

### 3.1. Antidiarrheal Effects

In the tropical and subtropical regions of the world, diarrhea is still one of the major causes of death. In developing countries, it is a principal cause of death in children under 5 years of age and the causes include infectious agents, plant toxins, and gastrointestinal disorders [130]. Many Western medicines, such as kaolin-pectin, bismuth, and loperamide, have long been used to alleviate the symptoms but have included undesirable side effects. It was reported that the ethanol extract of *A. paniculata* cured 88.3% of acute bacillary dysentery and 91.3% of acute gastroenteritis cases [91]. Administering andrographolide was reported to cure 91% of acute bacillary dysentery cases. The same cure rate (91.1%) was also achieved by administering a compound tablet containing andrographolide and neoandrographolide (at a ratio of 7 : 3) in cases of bacillary dysentery. This was reported to be higher than cure rates obtained with furazolidrne or chloramphenicol [91]. This compound has also been used traditionally to sluggish live as an antidote for colic dysentery and dyspepsia, and has been employed successfully in cases of general debility in convalescence after fever, livero disorders and advanced stages of dysentery. The juice of fresh leaves of *A. paniculata*, which generally contains andrographolide, is used as a domestic remedy to treat colic pain, loss of appetite, irregular stool, and diarrhea [131].

### 3.2. Anti-HIV Effects

Studies on the development of new anti-HIV drugs have begun worldwide in the past few years [132]. The growing incidence of drug-resistant HIV strains is one of the main problems in treating HIV infection, although current anti-HIV drugs can inhibit HIV infection. To avoid existing therapeutic difficulties, current searches for new anti-HIV agents are focused on discovering compounds with novel structures and different mechanisms of action [133]. Natural products and their derivatives have long been invaluable as a source of therapeutic agents for the development of medicine. The development of anti-HIV drugs derived from natural products is an area of research in which considerable effort should be dedicated in the future [134]. A clinical trial of andrographolide was conducted to examine 13 HIV-positive patients and five HIV-negative healthy volunteers. A planned protocol began with a dose of 5 mg/kg body weight for the first 3 weeks, increased to 10 mg/kg body weight for 3 weeks, and then increased to 20 mg/kg body weight for the final 3 weeks. Andrographolide administration significantly improved the CD4^+^ lymphocyte count from a baseline mean of 405 cells/mm^3^ to 501 cells/mm^3^ in HIV-positive patients. There was no statistically significant change in mean plasma HIV-1 RNA levels [3]. A recent study summarized that andrographolide derivatives may be promising candidates for preventing HIV infection [135], suggesting that andrographolide inhibited the gp120-mediated cell fusion of HL2/3 cells with TZM-bl cells.

### 3.3. Effects on Upper Respiratory Tract Infections

*A. paniculata *has been widely used for upper respiratory tract infections (URTIs). In a randomized, double-blind, and controlled study, Thamlikitkul et al. [73] administered *A. paniculata *at a dose of 6 g/day for 7 days to 152 Thai adults suffering from pharyngotonsillitis, and the efficiency has been reported to be similar to that of acetaminophen in relieving the symptoms of fever and sore throat. Cáceres et al. [136] clearly demonstrated that the treatment of *Andrographis paniculata* extract SHA-10 reduces the intensity of the symptoms of tiredness (OR = 1.28; 95% CI 1.07–1.53), sleeplessness (OR = 1.71; 95% CI 1.38–2.11), sore throat (OR = 2.3; 95% CI 1.69–3.14), and, HSP, (OR = 2.51; 95% CI 1.82–3.46) as compared with the placebo group in a duration-dependent manner. They have found that *Andrographis paniculata* extract treatment for 4 days significantly decreases in the intensity of all symptoms than in 2-day treatment group.

## 4. Dosage and Safety of Andrographolide

Numerous studies have been performed in different countries on the toxicity of *A. paniculata, *finding that it is extremely nontoxic, even at high doses (Table 2). Sakila et al. [137] conducted an antifertility study and found no toxicity, even at a high dose of *A. paniculata* that was administered to rats. The LD50 of andrographolide in male mice through the intraperitoneal route was reported to be 11.46 g/kg [138]. In a study of HIV-positive patients, a dose of 1,500–2,000 mg of andrographolide was administered daily for 6 weeks. The study was discontinued early despite some improvements in CD4^+^ counts [3], and the side effects were common. Intravenous administration of andrographolide (10 mg/kg) to rabbits showed no abnormal cardiovascular responses. Results from liver enzyme tests indicated that the heart, liver, kidney, and spleen of these rabbits were found to be normal [139]. Mice receiving an oral plant extract (10 g/kg) once a day for 7 days proved that no mortality was observed. In another test for toxicity, rats or rabbits receiving 1 g/kg of andrographolide orally showed no changes in body weight, blood count, or the functions of the liver, kidney, or other vital organs [94]. Singha et al. [56] noticed that pretreatment of *A. paniculata* and andrographolide at 500 mg/kg body weight and 125 mg/kg body weight, respectively, could minimize the toxicity when compared with the ethanol-treated group, as evidenced by different enzymatic assays in the liver and kidney tissues; the results were comparable with those of administering silymarin.

Our recent study show that andrographolide concentrations of 22 *μ*g/kg and 55 *μ*g/kg markedly lowered the mortality rate in mice challenged with ADP (700 mg/kg) from 90% to 60%, respectively, indicating that andrographolide effectively prevents thromboembolism (Table 1) [120]. Suo et al. [140] investigated the pharmacokinetics of andrographolide (10 mg/kg, i.v.) in rats and observed that the blood concentration of andrographolide was approximately 11 *μ*g/mL (approximately 30 *μ*M). Moreover, administering andrographolide causes no cytotoxic effects on platelets at concentrations between 35 and 150 mM [15]. Therefore, andrographolide is recommended to be clinically tested as a pharmaceutical agent.

## 5. Conclusion

Andrographolide, which exhibits notable pharmacological activities (Table 3), has attracted the interest of numerous researchers. Because of its rational activity, numerous andrographolide derivatives have been synthesized for the development of biological activities. Thus, this paper summarizes various experimental and clinical pharmacological activities of andrographolide, such as those that are antioxidant, anti-inflammatory, anticancer, antimicrobial and parasitic, hepatoprotective, antihyperglycemic, and antihypoglycemic. Evidence from clinical studies suggests that andrographolide reduces HIV symptoms, uncomplicated upper respiratory tract infections, including sinusitis and the common cold, and rheumatoid arthritis. Nevertheless, summarizing the effects on cardiovascular disease, NF-*κ*B, and platelet activation of this natural product is worthy of review, and additional studies must be conducted to confirm the toxicological properties of this novel molecule before taking place in clinical studies in patients. This summary offers pharmaceutical chemists and plant scientists additional thoughts for drug discovery. The combined drug discovery of andrographolide analogues will likely transform them into an effective assemblage of inflammation and cancer treatment in the future.

## Figures and Tables

**Table 1:** Effect of andrographolide on mortality of acute pulmonary thrombosis caused by intravenous injection of ADP in experimental mice.

	Total no. of mice	Number of deaths	Mortality rate (%)
Solvent control (0.5 DMSO)	6	0	0
ADP (700 mg/kg)	10	9	90
ADP (700 mg/kg) +			
andrographolide (*μ*g, kg)			
22	10	6	60*
55	10	5	50*

ADP: adenosine diphosphate; DMSO: dimethyl sulfoxide.

*P < 0.05 (compared with ADP control).

**Table 2:** Dosage and toxicity of *Andrographis paniculata* and its major natural product andrographolide.

Products name	Dosage/duration/route	Experimental models	Toxic effects	References
Andrographolide	10 mg/kg for 3 weeks	Human	No	[3]
Andrographolide	500 mg/kg bw for 7 days i.p.	Mice	No	[4]
Andrographolide	25–75 *μ*M	Platelets	No cytotoxicity	[15]
*A. paniculata *	20 mg/kg bw for 60 days, oral	Rats	No	[71]
Andrographolide	22–55 *μ*g/kg, i.v.	Mice	Lower mortality	[97]
*A. paniculata *	1 g/kg/day for 4, 6, and 8 weeks	Rats	No	[120]
Andrographolide	100 mg/kg, i.p.	Mice	No	[121]
Andrographolide	10 mg/kg, i.v.	Rats	No	[123]

i.p.: intraperitoneal; i.v.: intravenous; and bw: body weight.

**Table 3:** Experimental and clinical pharmacology of *Andrographis paniculata* and its major phytoconstituent andrographolide.

Pharmacological effects	Mechanisms	References
	(I) Experimental studies	

	↑ CAT, SOD, and GST;	
Antioxidant activity	↓ LDH	[23]
	↑ CAT, SOD, and GSH	[24]
	↓ TBARS	[26]

Anti-inflammatory effects	↓ LPS-induced NO production	[28–30]

Anticancer effects	↑ Cell differentiation	[35]
	↓ Proliferation of cancer cells	[3, 36]
	↑ IL-2 and IFN-c	[38]
	↓ Tumour growth	
	↓ Cell proliferation, migration, and cell cycle arrest at G2/M phase	[42]
	↓ E-selectin expression	[40]
	↓ Janus tyrosine kinases-signal transducers and activators of transcription, phosphatidylinositol 3-kinase and NF-*κ*B signalling pathways, suppression of hsp 90, cyclins, and cyclin-dependent kinases, MMPs and growth factors	
	↑ Tumor suppressor proteins p53 and p21	[41]

Immunomodulatory effect	↑ Antibody production	[43]
	↓ Delayed-type hypersensitivity response	
	↑ Proliferation of human peripheral blood lymphocytes	
	Key cytokines and the expression	[1, 2, 44]

	↓ ALT activity	[50]
Hepatoprotective effects	↓ Concanavalin A-induced liver injury and hepatocyte apoptosis	[52]
	↓ GOT, GPT, ACP, and ALP levels Losses of HBsAg, HBeAg, and HBV DNA	[54, 56]

Antimicrobial effects	Acted against herpes simplex virus 1 (HSV-1)	[59]
	Acted against nine bacterial strains such as	
	*Salmonella typhimurium, Escherichia coli, *	
	*Shigella sonnei, Staphylococcus aureus, *	[63]
	*Pseudomonas aeruginosa, Streptococcus pneumonia, *	
	*Streptococcus pyogenes, Legionella pneumophila,* and *Bordetella pertussis *	

	↓ Herpes simplex virus (HSV)	[62, 68]
Antiviral effects	Human immunodeficiency virus (HIV)	[3, 67]
	Flaviviruses and pestiviruses	[69]
	Dengue virus (DENV1)	[71]

Larvicidal and ovicidal effects	Affected the larval growth of *Anopheles stephensi *	[81]
	Ovicidal activity against various age groups of *Aedes Stephens *	[82]
	Larvicidal and ovicidal activities against *Culex quinquefasciatus* Say and *Aedes aegypti* L.	[83]

Renoprotective effects	↓ Gentamicin-induced increase in serum creatinine,	[86]
	serum urea, and blood urea nitrogen levels	

	↓ Spermatogenesis	[94]
Antifertility effects	↓ Degenerative changes in the seminiferous tubules, regression of Leydig cells, and regressive and/or degenerative changes in the epididymis, seminal vesicle,	[94]
	↓ ventral prostate, and coagulating glands	[94]

Antihyperglycemic activity	↓ TG	[24]
	↓ Blood glucose level	[99, 100, 102]

Hypolipidemic effects	↓ TC, TG, HDL-TC, and LDL-TC	[109]
	↓ Blood glucose, triglyceride, and LDL	[110]

Cardiovascular effects	Limiting blood flow to the brain, heart, and bodies of other organs	[112]
	Protect rat cardiomyocytes against	
	hypoxia injury by increasing GSH	[113]
	and antioxidant enzyme	
	↓ Coronary perfusion pressure	[114]

	↓ Platelet-activating factor (PAF)	[15]
Inhibitory effects on platelet aggregation	↑ eNOS-NO/cyclic GMP pathway	[120]
	↓ PLC*γ*2-PKC and PI3 kinase/Akt-MAPKs	

Inhibitory effects on NF-kappa B activation	↓ NF-*κ*B via the covalent modification of reduced Cys62 of p50	[13]
	↓ NF-*κ*B via blocking the binding of NF-*κ*B oligonucleotides to nuclear proteins	[128]

	(II) Clinical studies	

Anti-HIV effect	↑ CD4+ lymphocyte count	[3]
	↓ gp120-mediated cell fusion of HL2/3 cells with TZM-bl cells	[135]

Effects on upper respiratory tract infections	↓ Relieving the symptoms of fever and sore throat	[73]
	Tiredness, sleeplessness, sore throat, and nasal secretion	[136]

CAT: catalase; SOD: superoxide dismutase; GST: glutathione-S-transferase; LDH: lactate dehydrogenase; TBARS: thiobarbituric-acid-reactive substances; LPS: lipopolysaccharides; NO: nitric oxide; IL-2: interleukin-2; IFN-c: interferon-c; GOT: glutamate oxaloacetate transaminase; GPT: glutamate pyruvate transaminase; ALP: alkaline phosphatase; ACP: acid phosphatase; HBsAg: hepatitis B surface antigen; HBeAg: hepatitis B “e” antigen; ALT: alanine aminotransferase; TC: total cholesterol; TG: triglyceride; LDL: low-density lipoprotein; HDL: high-density lipoprotein; GHS: reduced glutathione; PLC-*γ*2: phospholipase C; PKC: protein kinase C; MAPK: mitogen-activated protein kinase; cGMP: cyclic guanosine monophosphate; eNOS: endothelial nitric oxide synthase; HSP: heat shock protein; MMP: matrix metalloproteinases.
